# MerTK Drives Proliferation and Metastatic Potential in Triple-Negative Breast Cancer

**DOI:** 10.3390/ijms25105109

**Published:** 2024-05-08

**Authors:** Mari Iida, Bridget E. Crossman, Kourtney L. Kostecki, Christine E. Glitchev, Carlene A. Kranjac, Madisen T. Crow, Jillian M. Adams, Peng Liu, Irene Ong, David T. Yang, Irene Kang, Ravi Salgia, Deric L. Wheeler

**Affiliations:** 1 Department of Human Oncology, University of Wisconsin-Madison, Madison, WI 53705, USA; iida@humonc.wisc.edu (M.I.); bridget.crossman@wisc.edu (B.E.C.); kkostecki@wisc.edu (K.L.K.); glitchev@wisc.edu (C.E.G.); ckranjac@mcw.edu (C.A.K.); mcrow3@wisc.edu (M.T.C.); jmadams7@wisc.edu (J.M.A.); 2 Departments of Biostatistics and Medical Informatics, University of Wisconsin-Madison, Madison, WI 53726, USA; pliu@biostat.wisc.edu (P.L.); irene.ong@wisc.edu (I.O.); 3 Carbone Cancer Center, University of Wisconsin-Madison, Madison, WI 53792, USA; 4 Department of Obstetrics and Gynecology, University of Wisconsin-Madison, Madison, WI 53705, USA; 5 Department of Pathology and Laboratory Medicine, University of Wisconsin-Madison, Madison, WI 53705, USA; dtyang@wisc.edu; 6 Department of Medical Oncology and Experimental Therapeutics, Comprehensive Cancer Center, City of Hope, Duarte, CA 91010, USA; ikang@coh.org (I.K.); rsalgia@coh.org (R.S.)

**Keywords:** MerTK, endoglin, TNBC, lung metastases

## Abstract

Triple-negative breast cancer (TNBC) is characterized by the absence of the estrogen receptor, progesterone receptor, and receptor tyrosine kinase HER2 expression. Due to the limited number of FDA-approved targeted therapies for TNBC, there is an ongoing need to understand the molecular underpinnings of TNBC for the development of novel combinatorial treatment strategies. This study evaluated the role of the MerTK receptor tyrosine kinase on proliferation and invasion/metastatic potential in TNBC. Immunohistochemical analysis demonstrated MerTK expression in 58% of patient-derived TNBC xenografts. The stable overexpression of MerTK in human TNBC cell lines induced an increase in proliferation rates, robust in vivo tumor growth, heightened migration/invasion potential, and enhanced lung metastases. NanoString nCounter analysis of MerTK-overexpressing SUM102 cells (SUM102-MerTK) revealed upregulation of several signaling pathways, which ultimately drive cell cycle progression, reduce apoptosis, and enhance cell survival. Proteomic profiling indicated increased endoglin (ENG) production in SUM102-MerTK clones, suggesting that MerTK creates a conducive environment for increased proliferative and metastatic activity via elevated ENG expression. To determine ENG’s role in increasing proliferation and/or metastatic potential, we knocked out ENG in a SUM102-MerTK clone with CRISPR technology. Although this ENG knockout clone exhibited similar in vivo growth to the parental SUM102-MerTK clone, lung metastasis numbers were significantly decreased ~4-fold, indicating that MerTK enhances invasion and metastasis through ENG. Our data suggest that MerTK regulates a unique proliferative signature in TNBC, promoting robust tumor growth and increased metastatic potential through ENG upregulation. Targeting MerTK and ENG simultaneously may provide a novel therapeutic approach for TNBC patients.

## 1. Introduction

Over two million new cases of breast cancer will be diagnosed in 2024, making it the most common type of cancer in women worldwide [1,2,3]. About 15–20% of breast cancers are characterized as triple negative. Triple-negative breast cancer (TNBC) is an aggressive subset of breast cancer that lacks expression of the estrogen receptor (ER), progesterone receptor (PR), and human epidermal growth factor receptor 2 (HER2) [4,5]. Because TNBC patients are not candidates for hormone receptor or HER2-targeted therapies, the current standard of care includes chemotherapy, radiation, and surgery. Patients diagnosed with early-stage disease exhibit a recurrence rate of around 50%, and patients with metastatic disease exhibit short progression-free survival after first-line therapy [6,7]. Understanding the molecular mechanisms by which TNBC gains its highly proliferative and metastatic properties is key to further identifying novel treatment strategies for these patients.

Receptor tyrosine kinases (RTKs) are a collection of 57 signaling proteins known to regulate many cellular processes, including growth, adhesion, migration, survival, and differentiation [8,9,10,11]. Expression of several RTKs has been implicated in the progression of TNBC, including EGFR, c-KIT, VEGF2, and PDGFRα; the most extensively studied of these candidates is EGFR [12,13,14,15,16,17]. The TAM (Tyro3, Axl, MerTK) family of RTKs has been shown to provide cancer cells with a strong advantage in proliferation and survival, leading to tumor progression [18,19]. MerTK is widely expressed in many cell types, including tumor cells and normal cells of hematopoietic lineage, and MerTK specifically has been implicated in the progression of many hematological and solid tumor malignancies [20,21]. Further, MerTK plays a key role in macrophage phagocytosis and contributes to homeostasis within the innate immune system, negatively regulating the release of pro-inflammatory signaling molecules and thereby preventing autoimmunity [21,22]. On the cancer cell, MerTK expression is associated with lower levels of apoptosis, increased proliferation, and cell cycle dysregulation [23,24,25].

In this study, we describe a link between MerTK, which is highly expressed in TNBC tumors, and cancer cell proliferation and tumor metastasis. Our data suggested that overexpressing MerTK in TNBC cell lines led to increased proliferation and robust in vivo tumor growth. Furthermore, these cell lines exhibited heightened migration/invasion potential in vitro and increased lung metastasis in vivo. In a model of MerTK overexpression, we identified the upregulation of endoglin (ENG), a small transmembrane receptor for TGFβ, as a key determinant of tumor metastasis in vivo. These findings suggest that MerTK overexpression could be creating favorable conditions for increased proliferation and metastasis through the upregulation of ENG in TNBC.

## 2. Results

### 2.1. MerTK Is Expressed in TNBC Preclinical Models

**Figure 1:**
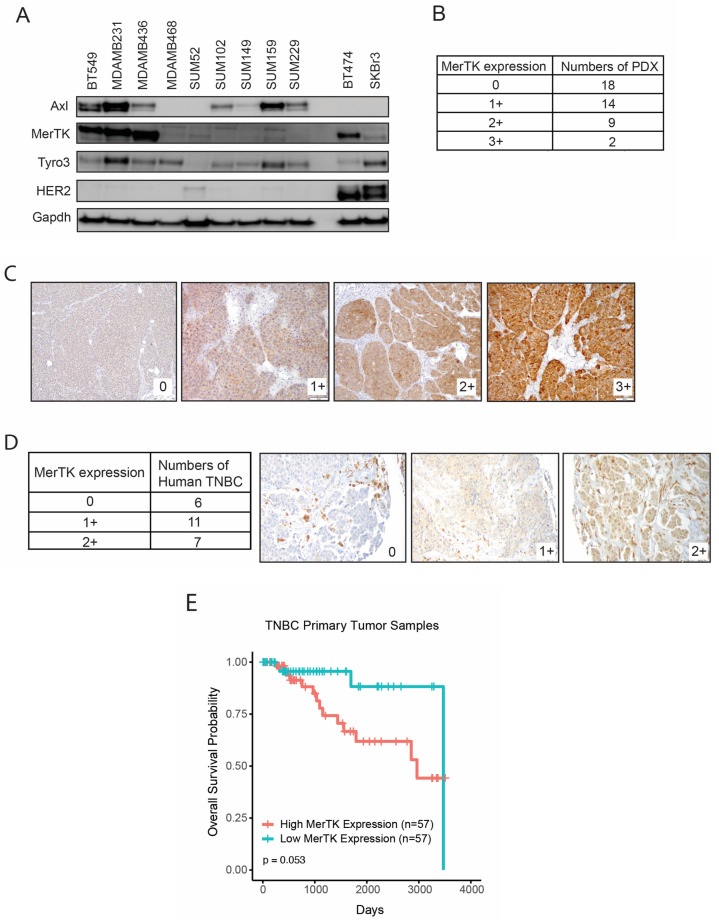
MerTK is expressed in Triple-negative breast cancer (TNBC) preclinical models, human tumors and associated with reduced overall survival. (**A**). Whole-cell lysate was harvested from nine TNBC cell lines and two human epidermal growth factor receptor 2 (HER2) positive breast cancer cell lines. The expression levels of Axl, MerTK, Tyro3, and HER2 were evaluated. Gapdh was used as a loading control. (**B**) MerTK expression levels in 43 TNBC patient-derived xenografts (PDX) Tissue Microarray (TMA) cores and individual PDX sections were measured using immunohistochemical staining (IHC). Representative images of low-to-high MerTK-expressing PDXs (**C**) are shown. Pathologic IHC quantitation (by D.T.Y) was determined using a categorical scale from 0 to 3+. Magnification ×20. (**D**) MerTK expression levels in 24 human TNBC specimens were evaluated using IHC. Pathologic IHC quantitation (by D.T.Y) was determined using a categorical scale from 0 to 2+. Magnification ×20. (**E**). A Kaplan–Meier analysis with a log-rank test for primary TNBC tumor samples in the Cancer Genome Atlas (TCGA) showed that high *MerTK* mRNA expression levels tended to be associated with reduced overall survival (OS). The 115 samples were stratified by MerTK level into two groups: 57 ‘high’ and 57 ‘low’, using one sample to define the median of MerTK level.

We previously reported that MerTK is expressed in various types of cancer, including TNBC [26]. To further evaluate MerTK expression levels in models of TNBC, we examined nine established TNBC cell lines and two HER2-positive cell lines by immunoblot analysis (Figure 1A, Supplemental Figure S8). Three TNBC cell lines—BT549, MDAMB231, and MDAMB436—exhibited high levels of MerTK expression, while three TNBC cells lines—MDAMB468, SUM52, and SUM159—had moderate MerTK expression, and three TNBC cell lines—SUM102, SUM149, and SUM229—had low MerTK expression levels. BT474, a HER2-positive breast cancer cell line, was also positive for MerTK expression. Next, we utilized a tissue microarray (TMA) composed of 43 human TNBC patient-derived xenograft (PDX) samples and stained for MerTK using standard immunohistochemical staining (IHC). An IHC analysis showed that MerTK is expressed in 58% (25/43) of PDXs, ranging from 0+ to 3+ (as scored by a board-certified pathologist (D.T.Y.)) (Figure 1B) and was localized predominantly to the cell membrane and cytoplasm (Figure 1C). Next, 24 TNBC patient samples were evaluated for MerTK expression by IHC, and our board-certified pathologist (D.T.Y.) scored it from 0 to 2+ (Figure 1D, Supplemental Table S3). Pathologic analysis indicated that 75% (18/24) of human TNBCs expressed MerTK. Four out of five normal/benign human breast tissues express very little MerTK (representative images are shown in Supplemental Figure S1). To further elucidate the role of MerTK in human TNBC, the correlation of *MerTK* RNA expression with overall survival (OS) was analyzed in 115 TNBC primary tumors from the Cancer Genome Atlas (TCGA). A Kaplan–Meier analysis with a log-rank test showed that TNBC patients with high *MerTK* expression exhibited worse OS but did not reach statistical significance (*p* = 0.053, Figure 1E) under a *p* < 0.01 cutoff. Collectively, these data demonstrate that MerTk is expressed in TNBC cell lines, PDX and human tumors.

### 2.2. MerTK Overexpression in TNBC Cell Lines Increases Cellular Proliferation and Tumor Growth

**Figure 2:**
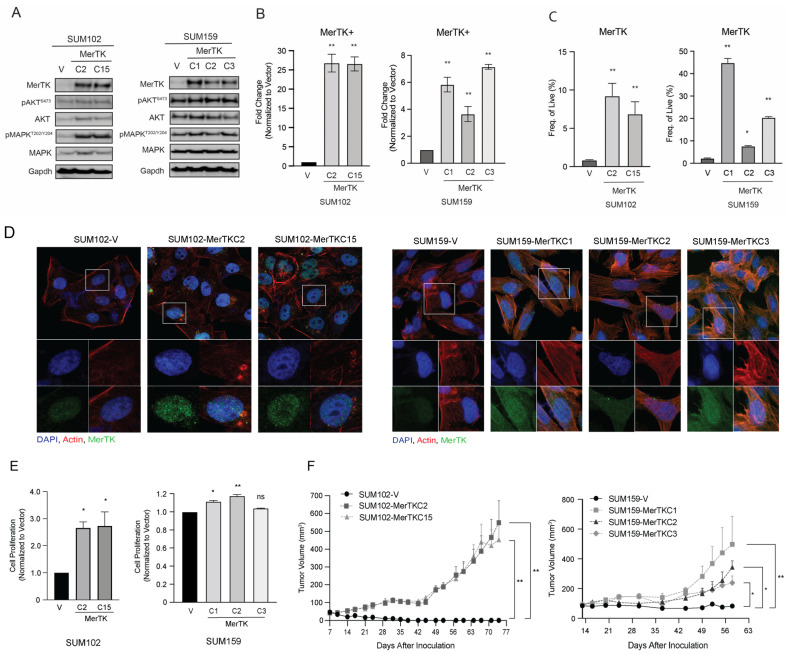
MerTK overexpression in TNBC cell lines increases cellular proliferation and tumor growth. (**A**). Whole-cell lysates were harvested from SUM102-MerTK and SUM159-MerTK clones as well as from their corresponding vector control clones. The indicated proteins were detected by immunoblot. Representative Gapdh was shown and used as a loading control. (**B**). RNA was harvested from SUM102-MerTK and SUM159-MerTK clones as well as from their corresponding vector control clones. After cDNA was synthesized, TaqMan qPCR assays were performed to detect *MerTK* mRNA expression levels. (**C**). The percentage of live cells expressing MerTK in SUM102-MerTK and SUM159-MerTK clones, as well as in their corresponding vector controls clones, was detected using flow cytometry. Data were analyzed using FlowJo. (**D**). Confocal microscopy was performed in SUM102-MerTK clones, SUM159-MerTK clones, and their corresponding vector control clones. Representative confocal images depict DAPI (blue), MerTK (green) and actin (red) staining. Magnification is 63×. Immunoblots, qPCR, flow cytometry, and IF are representative of two or three independent experiments. (**E**). Cell proliferation in SUM102-MerTK and SUM159-MerTK clones was measured via a crystal violet assay after 72 h. Mean values, SEs, and statistical analyses are representative of two independent experiments. *n* = 3, ns = not significant, * *p* < 0.05, ** *p* < 0.01. (**F**). SUM102-MerTK, SUM159-MerTK clones, and their corresponding vector control clones were injected into the flanks of athymic nude mice, and the tumor volume was measured twice weekly. *n* = 10.

To explore the role of MerTK in TNBC, two TNBC cell lines (SUM102 and SUM159) that have low endogenous MerTK expression were utilized to overexpress MerTK by stable transfection. This resulted in two MerTK-overexpressing clones in SUM102 (SUM102-MerTKC2 and SUM102-MerTKC15 clones) and three MerTK-overexpressing clones in SUM159 (SUM159-MerTKC1, SUM159-MerTKC2, and SUM159-MerTKC3 clones). MerTK overexpression in these clones as compared to their empty vector controls (SUM102-V or SUM159-V) was confirmed by immunoblot (Figure 2A and Supplemental Figure S6), quantitative PCR (Figure 2B, qPCR, SUM102-MerTK: ~25-fold, SUM159-MerTK: 3 to 7-fold), flow cytometry (Figure 2C, SUM102-MerTK: 6 to 9-fold, SUM159-MerTK: 7 to 45-fold), and confocal immunofluorescence (IF) (Figure 2D). To determine the impact of MerTK overexpression and activation in TNBC, we first examined cellular proliferation in SUM102-MerTK and SUM159-MerTK clones as compared to their vector controls using crystal violet assays. Both SUM102-MerTK clones (2- to 3-fold) and two out of three SUM159-MerTK clones (~1.2-fold) exhibited a significant increase in proliferation (Figure 2E). To determine the impact of MerTK overexpression in vivo, SUM102-MerTK and SUM159-MerTK clones were injected into both dorsal flanks of athymic nude mice (*n* = 5 mice/MerTK clone), and tumor growth was measured twice weekly for 70 days. The results of this mouse study indicated that SUM102-MerTK and SUM159-MerTK clones had the ability to grow in nude mice, whereas the vector clones did not (Figure 2F).

### 2.3. MerTK Overexpression in TNBC Cell Lines Increases Cell Migration, Invasion, and Metastatic Colonization of the Lung

**Figure 3:**
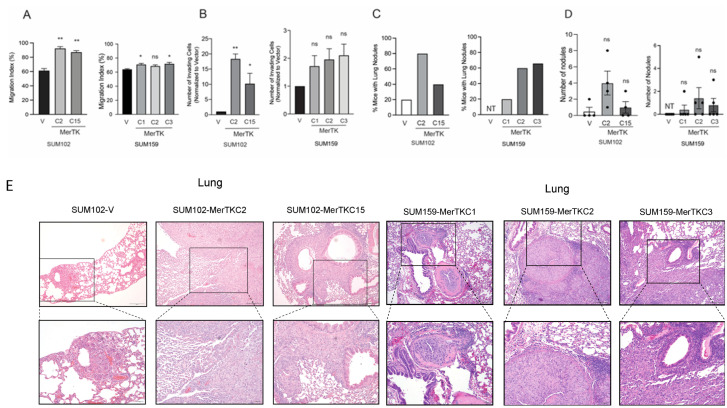
MerTK overexpression in TNBC cell lines increased cell migration, invasion, and metastatic colonization of the lung. (**A**). Cells were plated using a culture insert to create a cell-free gap. Gap width was measured immediately following culture-insert removal (0 h), 48 h later for SUM102-MerTK, and 24 h later for SUM159-MerTK (*n* = 3 in two independent experiments). (**B**). Cells were plated in a 24-well Boyden chamber with a Matrigel coating. Seventy-two hours later, invading cells in the bottom compartment of the chamber were stained with crystal violet. Images were taken, and invaded cells were counted (*n* = 3 in two independent experiments). All data points are presented as mean+/−SEM, ns = not significant, * *p* < 0.05, ** *p* < 0.01. (**C**). SUM102-MerTK and SUM159-MerTK clones, as well as their corresponding vector control clones, were injected into mice via the tail vein. Mice were sacrificed at day 118. The number of mice that had lung nodules were counted (*n* = 3~5). (**D**). The lungs were harvested, and the number of nodules was counted. (**E**). Lung tissues were fixed with 10% neutral buffered formalin followed by staining with hematoxylin and eosin (10× and 20× magnification). NT: no tumor developed.

It has been reported that MerTK promotes cell invasion in glioblastoma multiforme (GBM) and melanoma [21,27,28]. To investigate if MerTK influences cell migration and invasion in TNBC, migration assays were performed using MerTK-overexpressing SUM102 and SUM159 clones. A culture insert was used to create a cell-free gap of 500 μm (0 h). Gap width was measured at the indicated time points after the culture insert was removed. Compared to each vector control, both SUM102-MerTK clones (20–30%) and two out of three SUM159-MerTK clones (5–7%) displayed significantly faster gap closure after 24 or 48 h (Figure 3A, Supplemental Figure S2A). Next, the invasion potential of MerTK-overexpressing clones was measured using a transwell device coated with Matrigel. Both SUM102-MerTKC2 and MerTKC15 clones exhibited a significantly increased invasion capacity (10 to 18-fold) compared to SUM102-V clones after 72 h (Figure 3B, Supplemental Figure S2B). SUM159-MerTK clones exhibited increased cell invasion ~2-fold, although it was not significant compared to SUM159-V. Because the overexpression of MerTK in TNBC clones promoted increased cell migration and invasiveness, we hypothesized that MerTK could induce metastasis in vivo. To assess metastatic colonization, SUM102-MerTK and SUM159-MerTK clones were injected via the tail vein into nude mice. Mice injected with MerTK-overexpressing TNBC clones had more metastatic colonization of the lungs than those injected with the vector control after 118 days (Figure 3C). Eighty % (4/5) of mice and 40% (2/5) of mice had metastatic tumors in the lung in SUM102-MerTKC2 and MerTKC15 clones, respectively, whereas 20% (1/5) of mice had lung nodules in SUM102-V clones. Further, 20% (1/5), 60% (3/5) of mice, and 67% (2/3) of mice injected with SUM159-MerTKC1, SUM159-MerTKC2, and SUM159-MerTKC3 clones, respectively, had metastatic tumors in the lung, whereas no mice had lung nodules in the SUM159-V group. The lungs of mice injected with SUM102-MerTK or SUM159-MerTK clones exhibited metastatic infiltration with multiple nodules even though they did not reach statistical significance: 16 nodules in SUM102-MerTKC2, 4 nodules in SUM102-MerTKC15, 2 nodules in SUM159-MerTKC1, 7 nodules in SUM159-MerTKC2, and 4 nodules in SUM159-MerTKC3 in peri-bronchial interstitial tissues (Figure 3D). The lungs of one mouse injected with SUM102-V clones developed two alveolar nodules, and none of the mice injected with the SUM159-V clones developed nodules. A histological analysis showed that SUM102-MerTK cells that metastasized to the lung had more aggressive cytologic features, including nuclear pleomorphism and visible nucleoli, when compared to the SUM102-V lung metastasis (Figure 3E, Supplemental Figure S3). Further, no metastatic lesions in the liver were observed after gross examination, and body weight did not change in any group. Taken together, these results demonstrate that MerTK overexpression in TNBC promotes migration and invasion activities in vitro and in vivo.

### 2.4. MerTK Enhances Signaling Pathways Regulating Cell Cycle and Inhibits Those Regulating Apoptosis

**Figure 4:**
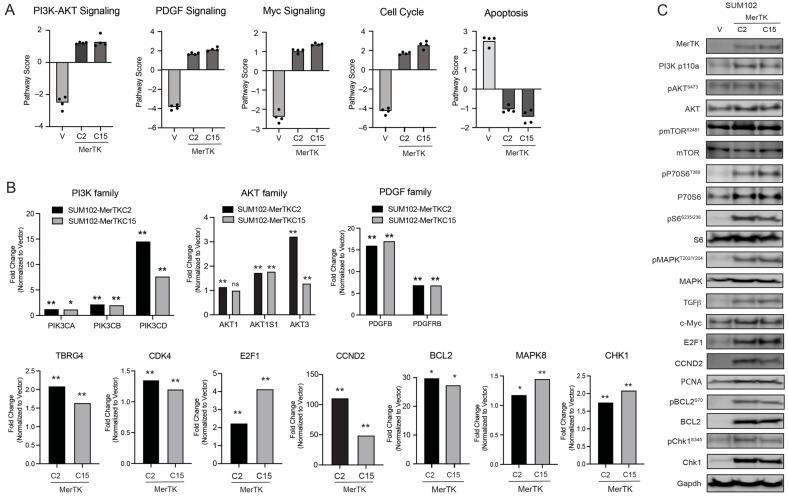
MerTK enhances several signaling pathways, progresses the cell cycle, and reduces apoptotic signaling. (**A**). The NanoString pathway score analysis showed that SUM102-MerTK clones obtained high or low scores in 5 signaling pathways (PI3K-AKT, PDGF, Myc, the cell cycle, and apoptosis) compared to SUM102-V clones. (**B**). The relative fold change of gene expression in members of each signaling pathway is shown in SUM102-MerTK clones following normalization to SUM102-V. *n* = 4. ns = not significant, * *p* < 0.05, ** *p* < 0.01. (**C**). Whole-cell lysates were harvested from SUM102-MerTK or SUM102-V clones. Immunoblotting was performed to detect indicated phospho- or total protein expression levels. Representative Gapdh was shown and used as a loading control.

Previous studies from our laboratory found that overexpression of MerTK activated P70S6K and C-RAF in head and neck squamous cell carcinoma (HNSCC) and TNBC cell lines [26]. To identify the specific pathways that were activated or regulated by MerTK, we profiled RNA from SUM102-MerTK clones using the NanoString Tumor Signaling 360 panel (NanoString Technologies, Seattle, WA, USA). Data were analyzed using the nSolver Advanced Analysis Package (nSolver v. 4.0, Advanced Analysis v. 2.0), and gene expression in SUM102-MerTK clones was compared to gene expression in SUM102-V clones. A pathway scoring analysis indicated that MerTK overexpression significantly impacts five pathway categories: PI3-AKT signaling, platelet-derived growth factor (PDGF) signaling, Myc signaling, the cell cycle, and apoptosis (Figure 4A). An analysis of gene expression fold change revealed that several key genes were upregulated in SUM102-MerTK clones (Figure 4B). *PI3K* (*PIK3Ca*, *PIK3CB*, and *PIK3CD*, 1.2- to 14.6-fold) and *AKT* (*AKT1*, *AKT1S1*, and *AKT3*, 1.2- to 3.2-fold) family genes were significantly upregulated in SUM102-MerTK clones. Two PDGF signaling-related genes (*PDGFB* and *PDGFRB*) were increased 6.9- to 17.0-fold in SUM102-MerTK clones. Genes involved in Myc signaling and cell cycle regulation were also upregulated (*transforming growth factor beta regulator 4*: *TBRG4* [~2-fold], *cyclin-dependent kinase 4*: *CDK4* [~1.3-fold], *E2F transcription factor 1*: *E2F1* [2- to 4-fold], and *cyclin D2*: *CCND2* [49- to 111-fold]) in SUM102-MerTK clones. Regulators of apoptosis, such as *BCL2* (~28-fold), *MAPK8* (~1.3-fold), and *checkpoint kinase 1* (*CHEK1*, ~2-fold), were increased in SUM102-MerTK clones. Immunoblot analysis was performed to verify and expand on the NanoString results (Figure 4C and Supplemental Figure S7). The phosphorylation of PI3K, AKT, mTOR, P70S6, S6, BCL2, MAPK, and Chk1 proteins and the expression of E2F1, CCND2, c-Myc, PCNA, and TGFβ proteins were increased in SUM102-MerTK clones compared with the SUM102-V control. Collectively, these results demonstrate that MerTK could enhance the cell cycle and inhibit apoptosis in TNBC clones expressing MerTK by a variety of pathways.

### 2.5. MerTK Facilitates Metastasis through Upregulation of Endoglin in TNBC

**Figure 5:**
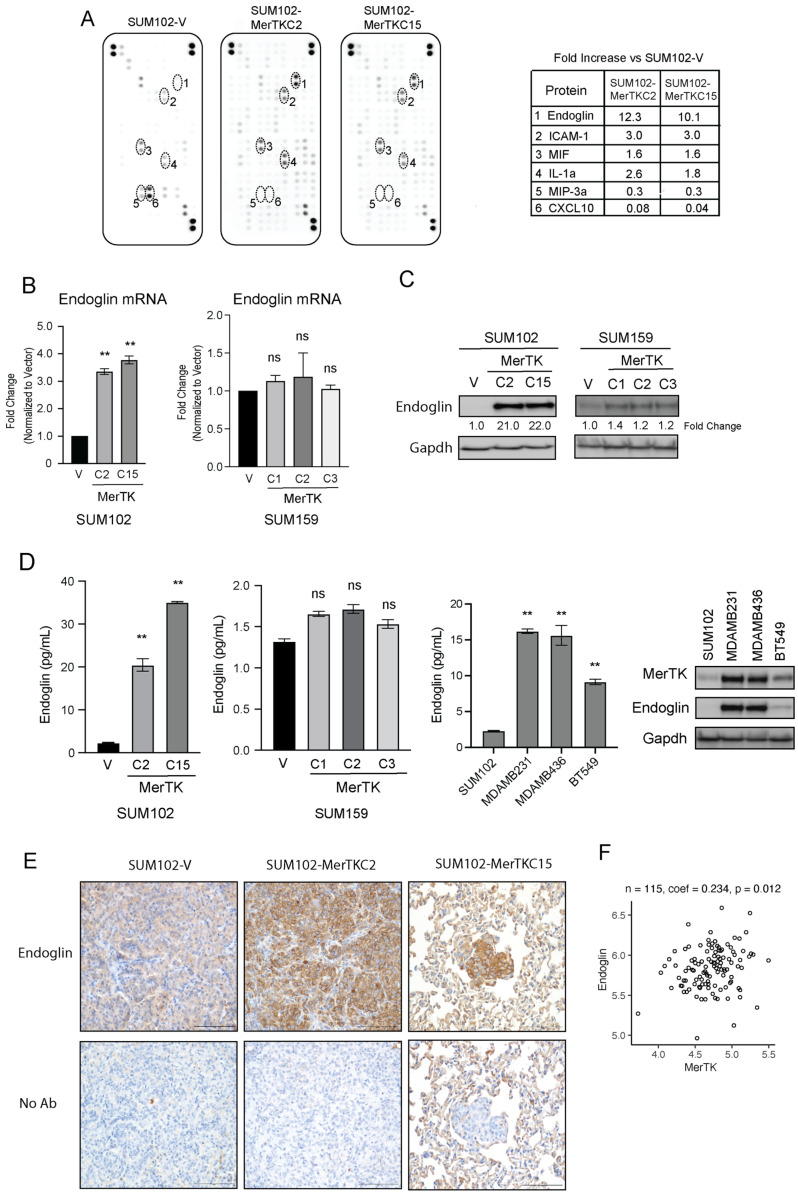
MerTK facilitates metastasis by enhancing endoglin (ENG) expression in TNBC. (**A**). The fold increase in the expression of cytokines in SUM102-MerTK or SUM102-V clones was determined using cytokine array. SUM102-V, SUM102-MerTKC2, and SUM102-MerTKC15 cell lysates were harvested and lysed with the extraction buffer provided as described according to the manufacturer’s instructions. The quantitation of protein levels was performed using ImageJ software (Ver. 1.53t). Data points are represented as the mean of duplicates. (**B**–**D**). RNA and whole-cell lysates were harvested from SUM102-MerTK, SUM159-MerTK, and their corresponding vector control clones as well as 3 TNBC cell lines (MDAMB231, MDAMB436, and BT549). TaqMan qPCR assays (**B**), immunoblotting (**C**), and ELISA (**D**) were performed to detect indicated protein and mRNA expression levels. Gapdh was used as a loading control. (**E**). Lung metastatic nodules from SUM102-MerTK clones expressed higher levels of ENG than the lung metastatic nodule from SUM102-V by IHC. Magnification ×40. (**F**). Analysis of 115 primary TNBC tumor samples in the TCGA showed a positive correlation (*p* = 0.012) between *MerTK* and *ENG* mRNA expression levels. ns = not significant, ** *p* < 0.01.

It has been reported that TNBC patients are susceptible to relapse with distant metastasis, and the median overall survival after a diagnosis of metastatic disease is 15 months [29,30]. We demonstrated that MerTK overexpression in TNBC clones promotes invasion and metastatic colonization of the lung (Figure 3); therefore, we hypothesized that MerTK may be increasing the production of growth factors and/or cytokines to influence metastatic potential in TNBC. To investigate this, we analyzed the levels of 102 different cytokines, chemokines, and growth factors in SUM102-MerTK clones using a cytokine array (R&D Systems, Inc. Minneapolis, MN). The results of this study showed that the levels of several cytokines were changed in SUM102-MerTK clones (Figure 5A). Notably, the protein expression of ENG was ~10-fold higher in SUM102-MerTK clones than the SUM102-V clone (Figure 5A). To validate this finding, we evaluated levels of ENG by qPCR (Figure 5B), immunoblot (Figure 5C), and ELISA (Figure 5D) in MerTK-overexpressing SUM102 and SUM159 clones. Just as in the cytokine array, SUM102-MerTK clones had increased expression of ENG mRNA (Figure 5B, ~3-fold) and protein (Figure 5C, ~20-fold and Figure 5D, 20- to 35-fold) as compared to the SUM102-V clone. Although the *ENG* gene expression levels in SUM159-MerTK clones did not increase significantly compared to SUM159-V (Figure 5B), ENG protein levels were ~1.3-fold increased by immunoblot (Figure 5C) and ELISA (Figure 5D) in SUM159-MerTK clones. Further, ELISA and immunoblot analyses were used to explore the expression levels of ENG and MerTK in three other TNBC cell lines, (MDAMB231, MDAMB436, and BT549) (Figure 5D). Interestingly, all three cell lines showed higher endogenous levels of MerTK and ENG compared to SUM102, which has low endogenous levels of both MerTK and ENG. These data demonstrate that TNBC cell lines and clones that either endogenously or exogenously express MerTK have higher levels of ENG expression. Because MerTK overexpression in TNBC clones caused increased lung metastasis (Figure 3), we evaluated ENG expression levels and localization in lung nodules. IHC analysis revealed strong ENG expression in the membrane and cytoplasm of tumor cells in the lungs of mice injected with SUM102-MerTK clones, whereas no ENG expression was observed in tumor cells in the lungs of mice injected with SUM102-V (Figure 5E, Supplemental Figure S4). In addition, analysis of 115 primary TNBC tumor samples from TCGA showed a positive correlation (Spearman’s rank correlation coefficient = 0.234, *p*-value = 0.012) between *ENG* and *MerTK* RNA levels (Figure 5F). Collectively, these results indicate that MerTK expression leads to increased ENG expression levels to promote TNBC metastasis.

### 2.6. MerTK Stimulates Endoglin to Promote Metastatic Activity in TNBC

**Figure 6:**
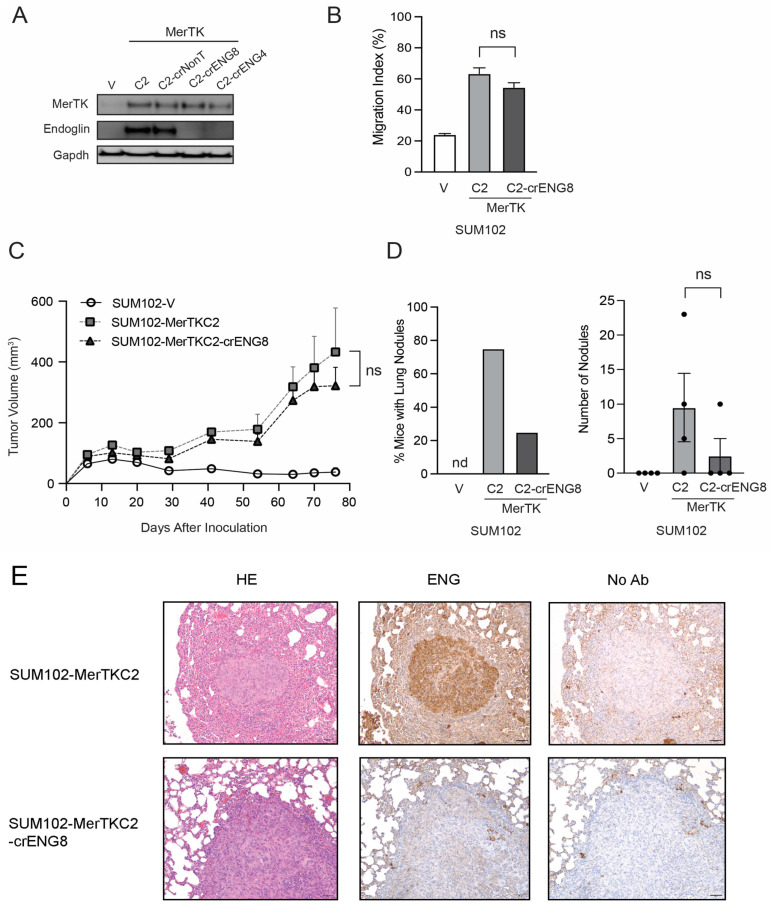
Endoglin leads to metastatic activity in TNBC. (**A**). Whole-cell lysates were harvested from SUM102-V, SUM102-MerTKC2, SUM102-MerTKC2-crNonT, SUM102-MerTKC2-crENG4, and SUM102-MerTKC2-crENG8 clones. Immunoblotting was performed to detect MerTK and ENG protein expression levels. Gapdh was used as a loading control. (**B**). Cells were plated using a culture insert to create a cell-free gap. The gap width was measured immediately following culture insert removal (0 h) and 30 h later (*n* = 3 in two independent experiments). (**C**). SUM102-V, SUM102-MerTKC2, and SUM102-MerTKC2-crENG8 cells (1 *×* 10^6^ cells) were injected into the flanks of athymic nude mice, and tumor volume was measured twice weekly. *n* = 5–10. (**D**). SUM102-V, SUM102-MerTKC2, and SUM102-MerTKC2-crENG8 cells were injected into mice via the tail vein. Mice were sacrificed at day 120 (*n* = 4). The lungs were harvested, and the number of metastatic nodules was counted. (**E**). Lung tissues were fixed with 10% neutral buffered formalin and stained with hematoxylin and eosin, and ENG was stained using IHC (*n* = 4, 20× magnification). ns = not significant.

**Figure 7:**
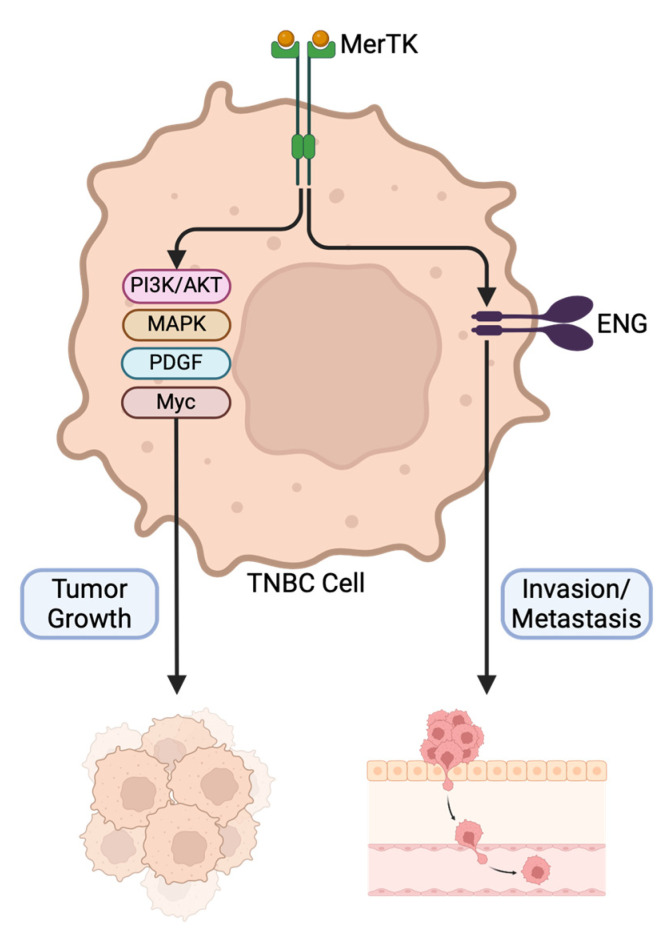
Two separate mechanisms for the role of MerTK and ENG in TNBC progression. MerTK drives cell proliferation and tumor growth via a network of pathways while ENG, whose expression is modulated by MerTK, promotes invasion and metastasis in TNBC.

Our findings indicated that MerTK increased ENG expression and enhanced lung metastatic potential. Thus, we hypothesized that both MerTK and ENG may be critical for TNBC metastasis. To test this hypothesis, we knocked out ENG in the SUM102-MerTKC2 clone via ribonucleoprotein (RNP)-mediated CRISPR technology (Figure 6A) and examined the cell migration and tumor growth of the resulting knockout clones (SUM102-MerTKC2-crENG). Similar to previous findings, SUM102-MerTKC2 showed increased migration potential (Figure 6B, ~3-fold) and faster tumor growth (Figure 6C) compared to SUM102-V at day 77. Interestingly, SUM102-MerTKC2 ENG knockout clone 8 (SUM102-MerTKC2-crENG8) exhibited similar migration (Figure 6B, ~2.8-fold) and tumor growth patterns (Figure 6C, Supplemental Figure S5) to SUM102-MerTKC2. In a model of metastasis, nearly 80% of mice had lung nodules after an injection of SUM102-MerTKC2 clones via the tail vein, and over 30 nodules in total were found after 120 days (Figure 6D). However, only 20% of mice had lung nodules after the SUM102-MerTKC2-crENG8 clones were injected, and the total number of lung nodules also decreased to 10; however, this decrease was not statistically significant. There were no lung nodules observed in mice injected with the SUM102-V clone. The expression of ENG in lung nodules injected with both SUM102-MerTKC2 and SUM102-MerTKC2-crENG8 clones was confirmed by IHC (Figure 6E). These data indicate that MerTK drives cell proliferation and tumor growth via a network of pathways while ENG, whose expression is modulated by MerTK, promotes metastasis but not tumor growth in TNBC (Figure 7).

## 3. Discussion

It is well described that TNBC tends to be more advanced at the time of diagnosis and more aggressive than ER- or PR-positive cancers [1]. Despite many studies proposing new treatment regimens and targets, it is still necessary to continue developing additional options, as these new treatments may not be effective in or recommended for all TNBC patients. Although aberrant MerTK expression has been found in many cancers, the mechanisms of increased cell proliferation and invasion driven by MerTK signaling in TNBC is still unclear. In this study, we investigated the role of MerTK in TNBC and demonstrated that MerTK drives cell proliferation and upregulation of ENG, which in turn increases TNBC metastatic potential.

Our previous study and several other reports have shown that MerTK is overexpressed in different types of cancer including non-small cell lung cancer [31,32,33,34], breast cancer [35,36], blood cancer [37,38], head and neck cancer [26,39], melanoma [28], leukemia [40], and glioblastoma [41]. Immunoblot analysis showed that ~67% of TNBC cell lines expressed some degree of endogenous levels of MerTK. (Figure 1A, Supplemental Figure S8). Our IHC results revealed that MerTK was expressed in ~60% of TNBC PDXs (Figure 1B) and 75% of human TNBC specimens (Figure 1D). Furthermore, TNBC patients whose tumor contained high *MerTK* RNA expression levels tended to have reduced OS (Figure 1D). In line with our results, several investigators reported that the genomic and gene expression profiles were very similar between PDXs and parental patient tumors [42,43,44,45]. Huelse et al. summarized that several other types of cancer, including lung, gastric, colorectal, and melanoma, also exhibit correlations between MerTK expression and poor prognosis [21]. MerTK has been noted to be expressed and play a role in TNBC [46]. When MerTK was overexpressed in TNBC cell lines (SUM102 and SUM159) with low endogenous levels of MerTK, these clones showed faster cell proliferation and increased migration/invasion compared to parental clones in vitro and in vivo (Figure 2 and Figure 3). The SUM102 cell line was derived from an early-stage breast tumor, and the SUM159 cell line was described as having carcinosarcomatous features [47]. Our tumor growth data demonstrated that overexpressing MerTK in the SUM102 cell line could promote the growth of early-stage TNBC tumors in nude mice. A histological analysis showed that SUM102-MerTK clones that metastasized to the lung had more aggressive cytologic features, including nuclear pleomorphism and visible nucleoli, when compared to the single vector control lung metastasis (Figure 3C,D). We speculate that this observation may also relate to the difference in metastatic distribution (alveolar vs. peri-bronchial interstitial tissue). Other investigators have also reported that the overexpression of MerTK promotes cancer cell migration [39,48]. Interestingly, Uribe and colleagues reported that Axl, but not MerTK or Tyro3, correlated with the migration and invasion of colorectal cells [49]. Further, Tyro3 was identified as a direct tumor suppressor miRNA gene target that regulates the proliferation, migration, and invasion of human hepatocarcinoma cell lines [50]. These observations suggested that each TAM family receptor plays an important role for cell migration and invasion in different cancers. Taken together, our data and others suggest that tumor expression of MerTK could be directing tumor cell proliferation, migration, and invasion in TNBC patients.

We utilized nCounter analysis to evaluate signaling pathways regulated by MerTK expression in TNBC. Several signaling pathways, including PI3k-AKT, PDGF, and Myc, were upregulated in SUM102-MerTK clones (Figure 4). These pathways drive cell cycle progression via the upregulation of E2F1, CCND2, C-Myc, and PCNA as well as reduce apoptosis via upregulation of BCL2, MAPK8, and CHEK1 in TNBC. Our lab previously reported that p70S6K, C-RAF, and AKT were activated in HNSCC when MerTK was overexpressed [26]. Another study using siRNA revealed that MerTK activated AKT and ERK in astrocytomas [51] and MerTK inhibitors decreased the phosphorylation of both AKT and ERK in GBM cell lines [41]. Linger et al. identified that MerTK knockdown reduced AKT, CREB, and BCL-XL expression levels and decreased survival signaling in NSCLC cells [24]. In line with other reports, the data we present here reveals further mechanistic insights into signaling pathways downstream of MerTK and expands the understanding of MerTK’s functions in TNBC.

MerTK has previously been implicated in cancer cell invasion and tumor metastasis [39,48]. Furthermore, ENG has been shown to mediate the invasion of angiosarcoma and colorectal cancer cells [52,53]. One of the most novel findings of our study was that ENG was upregulated in MerTK-overexpressing SUM102 and SUM159 clones and confirmed with a cytokine array, qPCR, immunoblot analysis, and ELISA (Figure 5). IHC analysis demonstrated that ENG was highly expressed in SUM102-MerTK metastatic tumor cells in the lung, whereas metastatic SUM102-V did not express ENG (Figure 5C). Interestingly, there seems to be a unique relationship between MerTK and ENG. Our results demonstrated that SUM102-MerTK clones with higher overexpression levels of MerTK showed a higher expression level of ENG compared to SUM159-MerTK, which has lower overexpression levels of both MerTK and ENG (Figure 5D). Further, three TNBC cell lines also showed higher endogenous levels of MerTK and ENG compared to SUM102, which has low endogenous levels of both MerTK and ENG. Thus, these data suggested that MerTK-overexpressing clones may create a positive feedback loop that maintains the expression of ENG to promote metastatic potential in TNBC. This hypothesis requires further investigation. Additionally, ENG expression levels were positively correlated with MerTK in the TCGA primary TNBC tumor samples (Figure 5F). These results suggested that MerTK could induce ENG expression in TNBC, and it would be important to evaluate the MerTK and ENG expression levels in TNBC patients for future metastatic potential. Interestingly, our NanoString nCounter and immunoblot analysis showed that TGFβ was overexpressed in SUM102-MerTK clones (Figure 4). TGFβ is a co-receptor of ENG and essential for angiogenesis as well as tumor vascularization [54,55]. Sakamoto et al. revealed that TGFβ induced overexpression of ENG in angiosarcoma cells [52], and we demonstrated here that MerTK induces overexpression of both ENG and TGFβ. Taken together, these results suggest that MerTK may upregulate ENG through TGFβ, though this mechanism is yet to be investigated. There is still debate as to the role of ENG as a tumor promoter or a tumor suppressor, with data suggesting that it may depend on the cancer cell type [56]. Our data suggest a role for ENG as a tumor promoter in TNBC.

Because the expression of MerTK and ENG increased lung metastasis in our TNBC model, we sought to determine whether ENG expression is critical for invasion/metastasis in TNBC. Although knocking out ENG in SUM102-MerTK clone did not affect cell migration and cell proliferation/tumor growth (Figure 6B,C), it did significantly reduce lung metastasis (Figure 6D). Tian et al. reported that ENG interacts with VEGFR2 and regulates the VEGF pathway in mouse endothelial cells and in vascular development both in vitro and in vivo [57]. They also showed that TRC105, an ENG-neutralizing antibody, enhanced anti-VEGF therapy. Additionally, there are several phase 1–2 clinical trials ongoing with TRC105 monotherapy or in combination with VEGF inhibitors [55]. Unfortunately, the phase 3 trial evaluating TRC105 in combination with Votrient (pazopanib)—a small molecule multi-kinase inhibitor that primarily inhibits VEGFRs, did not show greater benefit in patients with advanced or metastatic angiosarcoma [58]. A recent xenograft study showed that combination treatments with TRC105 and PD901 (anti-MEK) inhibited soft-tissue sarcomas and concluded ENG would be a promising therapeutic target [59]. Since targeting MerTK in TNBC with different inhibitors has shown promising results [21], evaluating therapeutic strategies simultaneously targeting MerTK and ENG may be a promising avenue for the treatment of TNBC patients.

In summary, our investigation revealed that MerTK regulates a unique proliferative signature, allowing for robust tumor growth and increased metastatic potential via the regulation of ENG. These results suggest that targeting MerTK and ENG simultaneously may be a viable therapeutic approach for TNBC patients.

## 4. Materials and Methods

### 4.1. Cell Lines

The human breast cancer cell lines BT549, MDAMB231, MDAMB436, MDAMB468, BT474, and SKBr3 were purchased from American Type Culture Collection (ATCC, Manassas, VA, USA). The human breast cancer cell lines SUM52, SUM102, SUM149, SUM159, and SUM229 were obtained from Asterand (Detroit, MI, USA). All cell lines were authenticated by the indicated source as well as by the TRIP lab at the University of Wisconsin-Madison (Madison, WI, USA) using short tandem repeat (STR) analysis and publicly available databases. All cell lines were maintained in their respective media (Corning, Corning, NY, USA) with 1% penicillin and streptomycin: BT549, MDAMB231, MDAMB436, SUM102, and SKBr3 in Dulbecco’s Modified Eagle’s Medium (DMEM) with 10% fetal bovine serum (FBS); BT474 in Roswell Park Memorial Institute (RPMI)-1640 with 10% FBS; SUM52, SUM149, SUM159, and SUM229 in Ham’s F-12 medium with 5% FBS, 1 mg/mL hydrocortisone, and 5 mg/mL insulin; and MDAMB468 in DMEM/F12 1:1 medium with 10% FBS.

### 4.2. Plasmid Constructs, Transfection, and CRISPR Technology

pDONR223-MERTK (RRID:Addgene_23900) was a gift from William Hahn and David Root, and *MerTK* was subcloned into the BamHI/NotI restriction sites of the pcDNA6.0 expression vector (Life Technologies, Carlsbad, CA, USA). Transfection was performed using Lipofectamine3000 and Opti-MEM (Life Technologies) according to the manufacturer’s instructions. Antibiotic selection was started 48 h after transfection via addition of blasticidin (1 µg/mL) to the growth media. For CRISPR editing, SUM102-MerTKC2 cells (3 × 10^4^ cells) were transfected with two predesigned TrueGuide sgRNA targeting *endoglin* (CRISPR768842 and CRISPR1060035, Invitrogen, Waltham, MA, USA) or TrueGuide sgRNA Negative Control (#A35526) using Invitrogen TrueCut Cas9 v2 and Lipofectamine CRISPRMAX (Invitrogen) according to the manufacturer’s recommendations. Two days after transfection, cells were harvested and stained with MerTK (APC anti-human mouse MerTK #367612, BioLegend, San Diego, CA, USA) as well as endoglin (PE anti-endoglin, #800504, BioLegend) antibodies. Then one cell per well was sorted in a 96-well plate by BD FACSAria Cell Sorters (BD, Franklin Lakes, NJ, USA) for clonal isolation and expansion.

### 4.3. Cell Proliferation, Wound Migration Model, and Invasion Assay

Crystal violet assays were used to determine relative numbers of viable cells 72 h after plating. Culture inserts (#81176 Ibidi, Gräfelfing, Germany) were used to measure cell migration. Cells (5 × 10^4^/mL, 70 μL) were applied to each well of the culture insert. After 24 h, a cell-free gap of 500μm was created by removal of the culture insert. The cells were incubated at 37 °C for an additional 24–48 h. Images were captured every 6 h using an inverted phase-contrast microscope with ThorCam software (Ver.3.2.1, Thorlabs Inc., Newton, NJ, USA). The percentage of gap closure in three randomly chosen fields was calculated. Invasion assays were performed using 24-well transwell inserts with a transparent PET plastic membrane (8 µm pore size). Membranes were coated with Matrigel (R&D Systems, Minneapolis, MN, #3433-005-01, diluted 1:50 with PBS) for 24 h before experiments [60]. An amount of 5 × 10^4^ cells were plated to each well of transwell insert. After 72 h of incubation, the bottom of chambers were stained with crystal violet. For quantitative evaluation, cell numbers in the chambers were counted in 3 randomly selected areas.

### 4.4. Immunoblotting

Whole-cell protein lysis, protein quantification, and immunoblot analysis were performed as described previously [61,62]. Antibodies were used according to the manufacturer’s instructions (Supplementary Table S1).

### 4.5. Xenograft Flank Models and Tail Vein Injection

Female athymic nude mice (4–6 weeks old) were obtained from Envigo (Indianapolis, IN, USA). Animal procedures and maintenance were conducted in accordance with institutional guidelines of University of Wisconsin-Madison. Cells (1 × 10^6^ cells) were resuspended in PBS with Matrigel (50% *v*/*v*, R&D Systems, #3433-005-01) and inoculated via subcutaneous injection into the dorsal flank of each mouse, and tumor volume was measured using a digital caliper. Single-cell suspensions of tumor cells were prepared prior to tail vein injection. Mice were intravenously (IV) injected with 1 × 10^6^ cancer cells in 100 μL PBS in the tail vein using insulin syringes.

### 4.6. Immunohistochemistry (IHC)

PDX tumors were obtained from Patient-Derived Xenograft Core, Baylor College of Medicine, and Jackson Laboratory (Bar Harbor, ME, USA). Human TNBC and normal/benign human breast tissues were obtained from Translational Research Initiatives in Pathology (TRIP)lab, UWCCC. PDX tumors, normal/benign human breast tissues, and the TNBC cancer patient TMA were stained using the Universal Quick Kit (#PK-8800, Vector Laboratories, Newark, CA, USA) according to manufacturer’s recommendation. Sections were heated in TRIS-EDTA buffer (pH 9.0) in a decloaking chamber and incubated overnight at 4 °C with MerTK (1:50, ab52968, Abcam, Cambridge, UK) or endoglin (1:250, ab169545, Abcam) antibodies or with no antibody as a control. Antibody binding was detected using 3,3′-diaminobenzidine substrates (Vector Laboratories) and counterstained with Mayer’s hematoxylin (Thermo Fisher Scientific, Waltham, MA, USA). Samples were examined using an Olympus BX51 microscope. Images are shown at a magnification of 10×, 20×, or 40×.

### 4.7. Immunofluorescent Staining (IF)

SUM102 and SUM159 cells were plated on Millicell EZ slide 4-well glass chamber slides (Millipore Sigma, St. Louis, MO, USA), allowed to attach over 24 h, fixed in 3% paraformaldehyde, and permeabilized in 0.3% Triton X-100. Cells were blocked in 5% goat serum and incubated overnight at 4 °C with MerTK primary antibody (Santa Cruz Biotechnology, Dallas, TX, USA, #365499, 1:100). Antibody binding was detected with mouse Alexa Fluor 488 secondary antibody (1:400, #A-11001, Invitrogen), actin was stained with ActinRed 555 (1:400, #R37112, Invitrogen), and nuclei were stained with DAPI (#D1306, Thermo Fisher Scientific). Samples were imaged using a Leica SP8 STED Confocal/Super-Resolution microscope. Images are shown at a magnification of 63×.

### 4.8. RNA Analysis

Total RNA from cells was isolated with RNeasy Mini kit (Quiagen, Germantown, MD, USA) according to manufacturer’s recommendation. After quantification of RNA by a Nano-drop spectrophotometer (Thermo Fisher Scientific), cDNA was synthesized using qScript cDNA SuperMix (Quantabio, Beverly, MA, USA). qPCR was conducted using the CFX96 Real-Time PCR System (Bio-Rad, Hercules, CA, USA). TaqMan Fast Advanced Master Mix and TaqMan probes (Thermo Fisher Scientific) were used (Supplemental Table S2). All reactions were performed in quadruplicate and repeated two to three times. The levels of gene expression were analyzed using the ΔΔCT method. Gapdh and 18S rRNA were used as normalization controls.

NanoString nCounter Tumor Signaling 360 panel on an nCounter MAX system was also used to analyze RNA expression levels. The UW-Madison TRIP lab performed sample preparation, hybridization, and scanning on the nCounter Digital Analyzer. Quality control was performed, and fold change data for each gene were obtained from RNA counts of *n* = 4 samples per group using the nSolver 4.0 software. Pathway scores were obtained from raw count data by selecting the SUM102-V samples as a covariate and SUM102-MerTKC2 and SUM102-MerTKC15 samples as variables using the Pathway Scoring Module in the Advanced Analysis 2.0 add-on to the nSolver software.

### 4.9. Flow Cytometry

Cells were harvested and processed as previously described [63]. Cells were analyzed using an Attune NxT flow cytometer (Thermo Fisher Scientific). MerTK expression levels were analyzed using FlowJo software (Ver. 10, BD, Franklin Lakes, NJ; RRID:SCR_008520). Antibodies were used according to the manufacturer’s instructions (Supplementary Table S1).

### 4.10. Cytokine Array

SUM102-V- and SUM102-MerTK-overexpressing cell lines (SUM102-MerTKC2 and SUM102-MerTKC15) were analyzed using the Human XL Cytokine array kit (#ARY222B, R&D systems). Cell lysates were incubated with the membrane according to the manufacturer’s protocol. The relative protein expression level was compared following quantification of digital images in ImageJ (Ver. 1.53t, RRID:SCR_003070).

### 4.11. TNBC Primary Tumor for Analysis

*MerTK* and *endoglin* mRNA expression levels as well as tumor subtypes were downloaded from the Cancer Genome Atlas (TCGA) (https://portal.gdc.cancer.gov/, The TCGA RNA expression data was downloaded on 7 April 2017 and the tumor subtype data was downloaded on 17 December 2018). TNBC tumors were identified by their negative ER, PR, and HER2 IHC status.

### 4.12. Statistical Analysis

Statistical analyses were performed using Prism 10 (GraphPad Prism, RRID:SCR_002798) and R. Differences were considered significant when *p* < 0.05.

## Data Availability

The datasets used and/or analyzed during the current study are available from the corresponding author on reasonable request.

## Supplementary Materials

The following supporting information can be downloaded at: https://www.mdpi.com/article/10.3390/ijms25105109/s1.
