# Supplementary material for: MerTK Drives Proliferation and Metastatic Potential in Triple-Negative Breast Cancer
Source: Int J Mol Sci. 2024 May 8;25(10):5109. doi: 10.3390/ijms25105109 (PMC11121248; doi:10.3390/ijms25105109)
Supplement: Supplementary file 1 [file ijms-25-05109-s001.zip › ijms-2905834-supplementary.pdf]

Supplemental Figure S1. MerTK expression level in normal/benign human breast tissues. Magnification x20

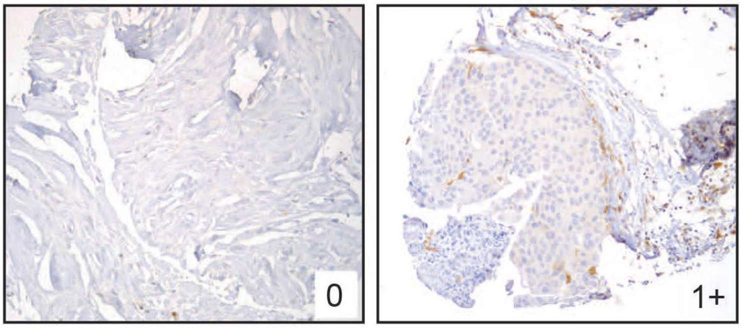

Supplemental Figure S2. The representative pictures of cell migration and invasion. Arrows indecated cells that invated through matrigel membranes.

A. Migration

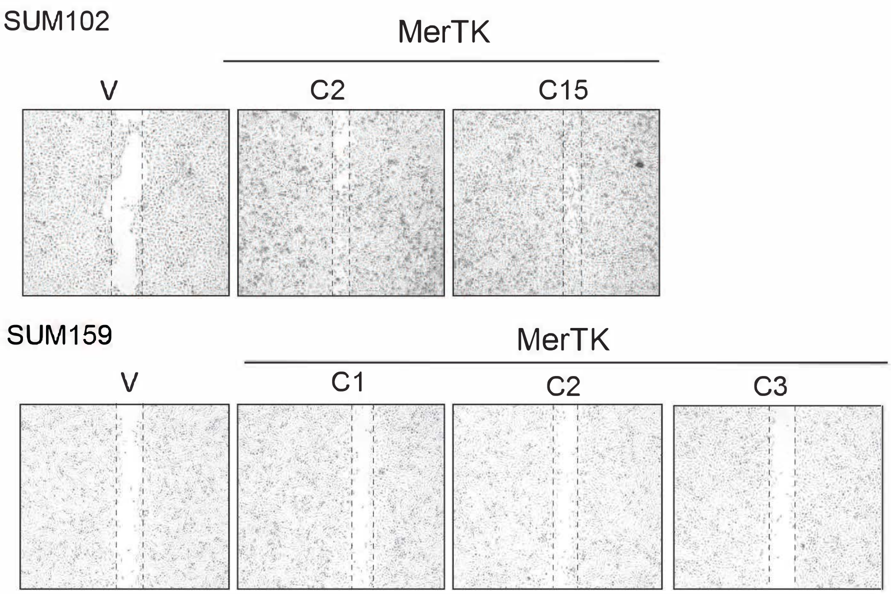

B. Invasion

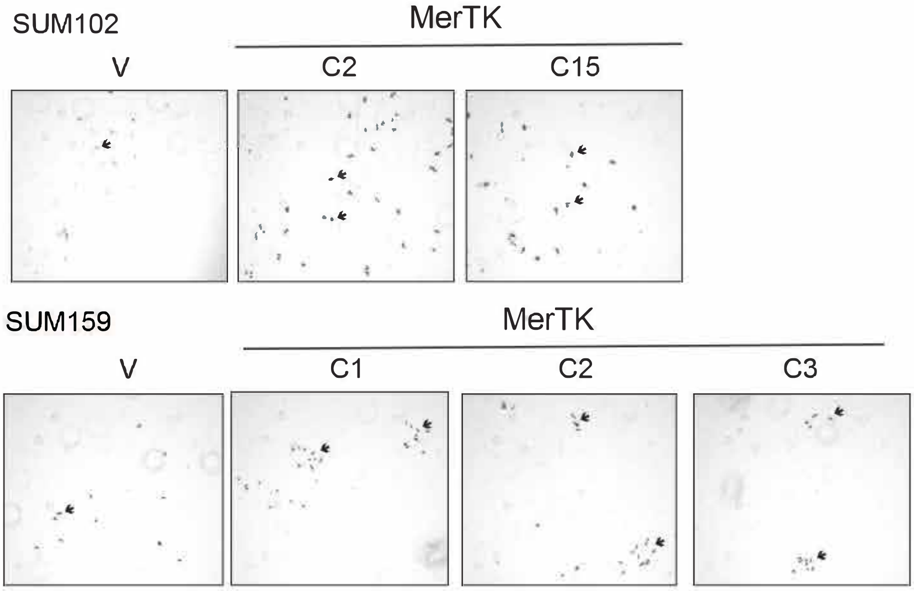

Supplemental Figure S3. Representative photographs of the lung with metastatic nodules are shown (arrow) in SUM159-MerTK clones

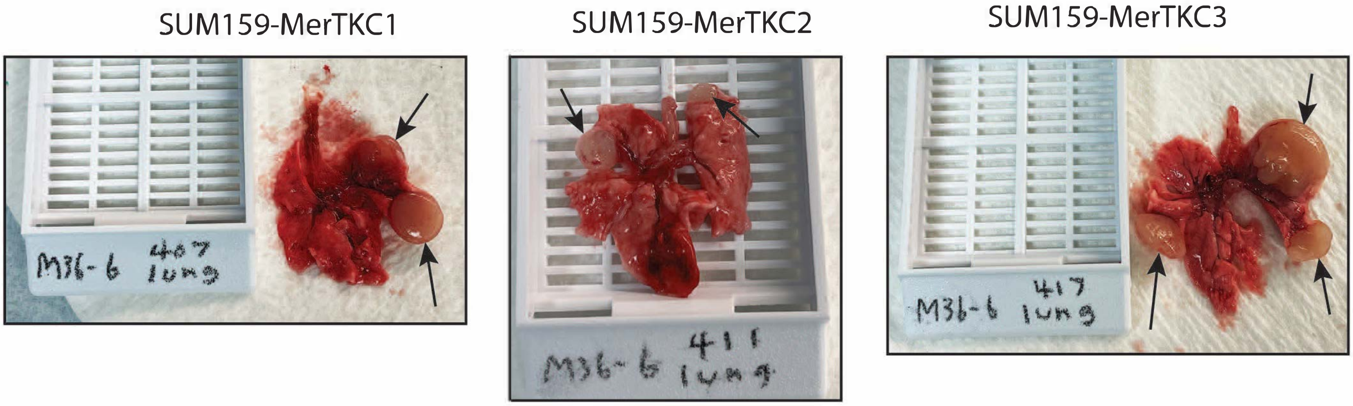

Supplemental Figure S4. Expression of ENG in lung metastatic nodules from SUM102-MerTK clones was analyzed by IHC. Magnification x40.

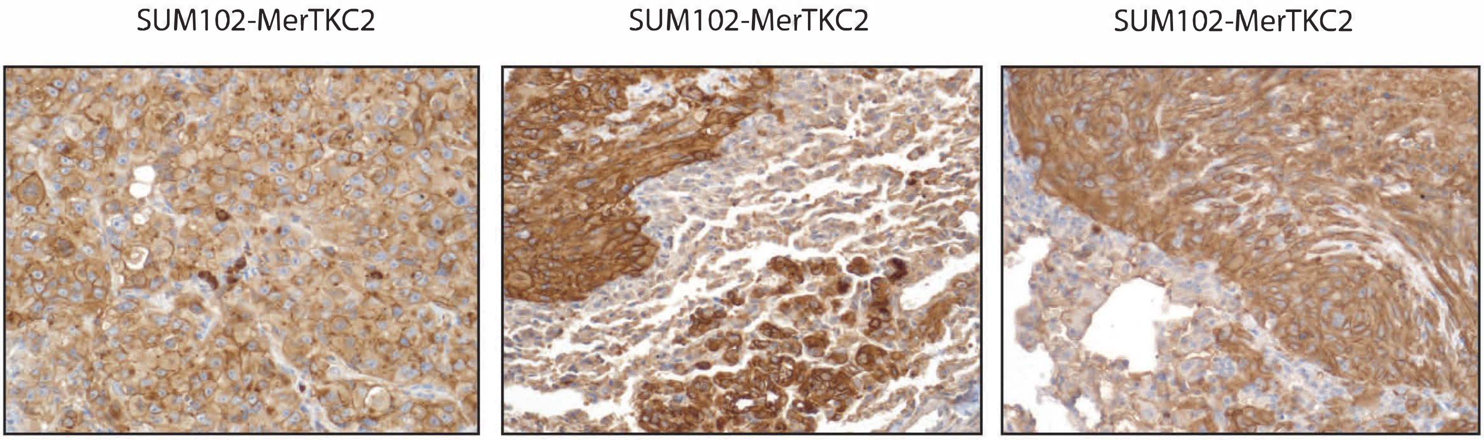

Supplemental Figure S5. Tumor weights in SUM102-V, SUM102-MerTKC2, and SUM102-MerTKC2-crENG8 cells. Tumor weight was measured at the tumor collection. (*n*=5-10). *P < 0.05.

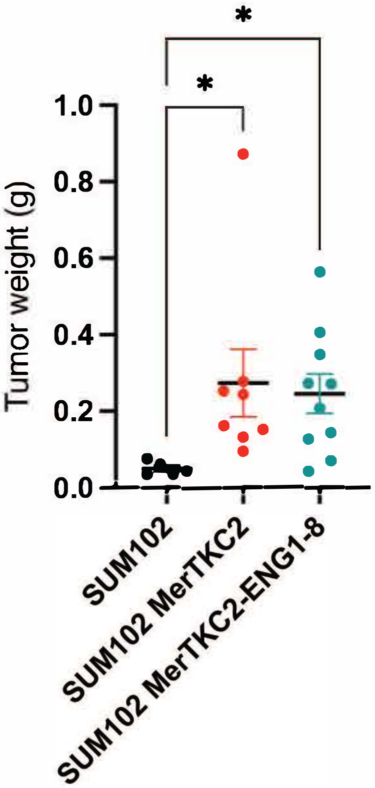

Supplemental Figure S6. Cell lysates from SUM102-MerTK and SUM159-MerTK clones, as well as from their corre-sponding vector control clones were analyzed by Western blotting with antibodies against MerTK, p-AKT (Ser 4 73), AKT, p-MAPK, MAPK,and Gapdh. Each Gapdh is shown as a loading control.

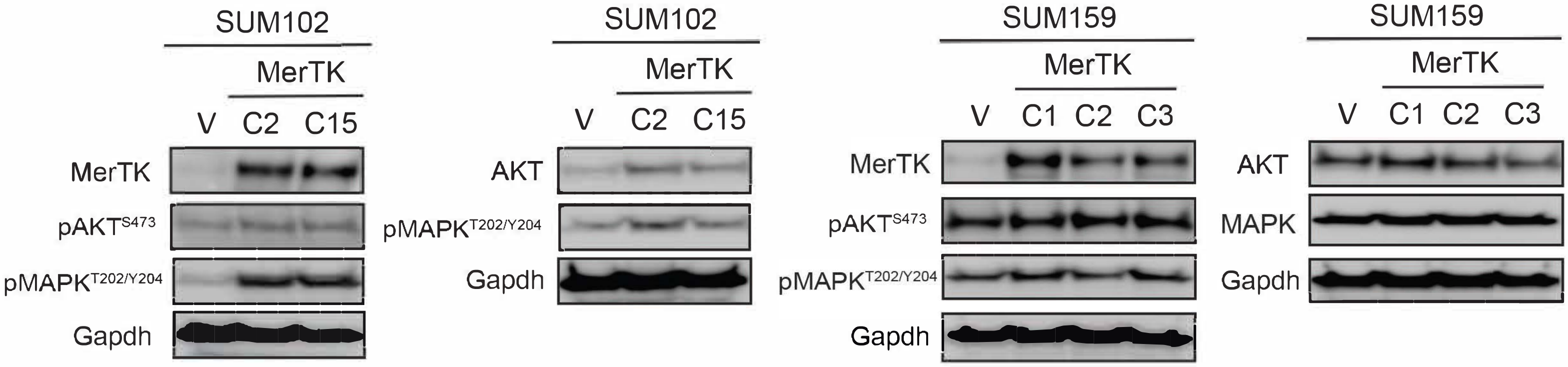

Supplemental Figure S7. Cell lysates from SUM102-MerTK and vector control clone were analyzed. The indicated proteins were detected by Immunoblot. Each Gapdh is shown as a loading control.

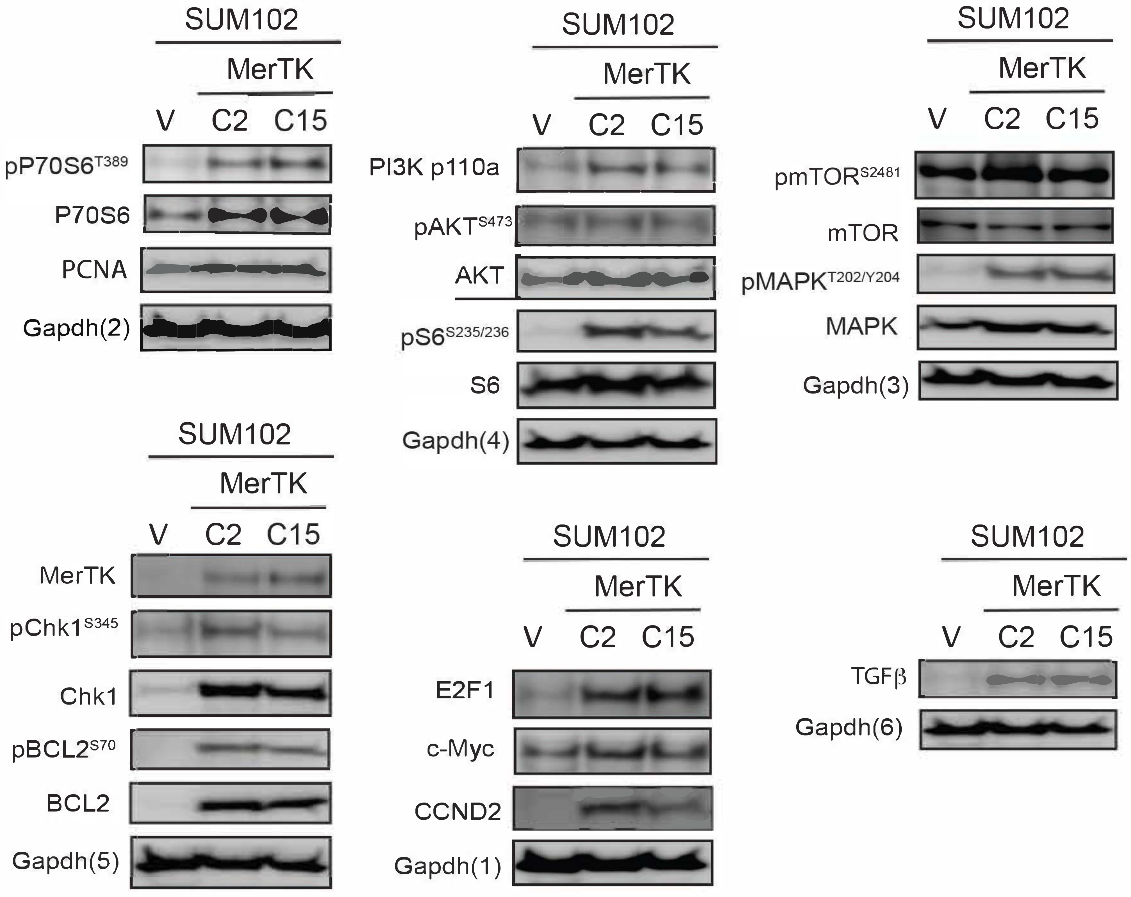

Supplemental Figure S8

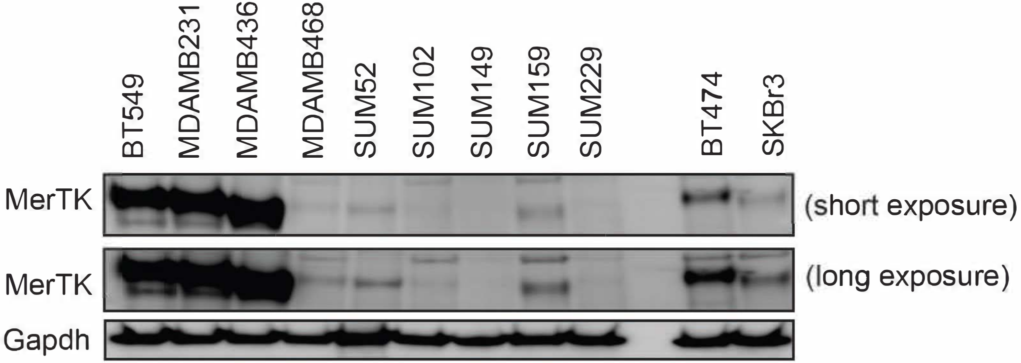

Supplemental Table S1: Immunoblot, Flow cytometry and Immunohistochemistry (IHC) Antibodies

Antigen	Vendor	Catalog Number, dilution
Axl	Cell Signaling Technologies, Danvers, MA, USA	8661, 1:1000
MerTK (Immunoblot)	Cell Signaling Technologies, Danvers, MA, USA	4319, 1:1000
MerTK (IHC)	Abcam, Cambridge, United Kingdom	Ab52968, 1:50
MerTK (Flow)	BioLegend, San Diego, CA, USA	367612
Tyro3	Cell Signaling Technologies, Danvers, MA, USA	5585, 1:1000
GAPDH	Cell Signaling Technologies, Danvers, MA, USA	2118, 1:3000
Endoglin (Immunoblot)	Cell Signaling Technologies, Danvers, MA, USA	14606, 1:1000
Endoglin (IHC)	Abcam, Cambridge, United Kingdom	Ab169545, 1:250
Endoglin (Flow)	BioLegend, san Diego, CA, USA	800504
Akt	Cell Signaling Technologies, Danvers, MA, USA	2920, 1:1000
pAkt	Cell Signaling Technologies, Danvers, MA, USA	4060, 1:1000
PI3K p100α	Cell Signaling Technologies, Danvers, MA, USA	4255, 1:1000
pmTOR S2481	Cell Signaling Technologies, Danvers, MA, USA	2974, 1:1000
mTOR	Cell Signaling Technologies, Danvers, MA, USA	2972, 1:1000
pP70S6 T389	Cell Signaling Technologies, Danvers, MA, USA	9234, 1:1000
P70S6	Cell Signaling Technologies, Danvers, MA, USA	9202, 1:1000
pS6 S235/236	Cell Signaling Technologies, Danvers, MA, USA	4856, 1:1000
S6	Cell Signaling Technologies, Danvers, MA, USA	2317, 1:1000
pBCL2 S70	Cell Signaling Technologies, Danvers, MA, USA	2827, 1:1000
BCL2	Cell Signaling Technologies, Danvers, MA, USA	15071, 1:1000
E2F1	Cell Signaling Technologies, Danvers, MA, USA	3742, 1:1000
pMAPK T202/Y204	Cell Signaling Technologies, Danvers, MA, USA	9101, 1:1000
MAPK	Cell Signaling Technologies, Danvers, MA, USA	9107, 1:1000
Cyclin D2	Cell Signaling Technologies, Danvers, MA, USA	3741, 1:1000
TGFβ	Cell Signaling Technologies, Danvers, MA, USA	3709, 1:1000
HER2	Cell Signaling Technologies, Danvers, MA, USA	4290, 1:1000
Live/Dead GhostRed 780 (Flow)	Tonbo Biosciences, San Diego, CA, USA	13-0865-T100

Supplemental Table S2: TaqMan Probes

Gene	Assay ID
Human MerTK	Hs01031979_m1
Human Endoglin	HS00923996_m1
Human ACTB	4332645
Human 18S	4332641

Supplemental Table S3: TNBC patient's clinical data

Subject	Stage	Histology category	ER (Clinical Data)	PR (Clinical Data)	HER2 (Clinical Data)	MerTK expression
TNBC 1	IIB	ductal	NEG	NEG	NEG	2+
TNBC 2	IIA	ductal	NEG	NEG	NEG	1+
TNBC 3	IIA	ductal	NEG	NEG	NEG	1+
TNBC 4	I	mammary	NEG	NEG	NEG	1+
TNBC 5	IIA	ductal	NEG	NEG	NEG	2+
TNBC 6	I	ductal	NEG	NEG	NEG	2+
TNBC 7	IIA	ductal	NEG	NEG	NEG	1+
TNBC 8	IIIC	ductal	NEG	NEG	NEG	1+
TNBC 9	IIA	ductal	NEG	NEG	NEG	2+
TNBC 10	IIA	ductal	NEG	NEG	NEG	1+
TNBC 11	IIA	ductal	NEG	NEG	NEG	0
TNBC 12	IIA	ductal	NEG	NEG	NEG	1+
TNBC 13	II	ductal subtype	NEG	NEG	NEG	0
TNBC 14	IIA	ductal	NEG	NEG	NEG	1+
TNBC 15	IIB	mammary	NEG	NEG	NEG	2+
TNBC 16	IIIA	ductal	NEG	NEG	NEG	2+
TNBC 17	IIA	ductal	NEG	NEG	NEG	1+
TNBC 18	IIB	ductal	NEG	NEG	NEG	1+
TNBC 19	III	ductal subtype	NEG	NEG	NEG	1+
TNBC 20	IIIA	ductal	NEG	NEG	NEG	0
TNBC 21	IIIC	ductal	NEG	NEG	NEG	0
TNBC 22	IIB	ductal	NEG	NEG	NEG	0
TNBC 23	IIB	ductal	NEG	NEG	NEG	2+
TNBC 24	IIB	ductal	NEG	NEG	NEG	0
